# Taming wild genomes: perspectives on mechanisms governing differential introgression in exotic rice germplasm and their applications in breeding

**DOI:** 10.3389/fpls.2025.1489244

**Published:** 2025-01-22

**Authors:** Avinash Shrestha, Christian J. Stephens, Rosalyn B. Angeles-Shim

**Affiliations:** Department of Plant and Soil Science, Davis College of Agricultural Sciences and Natural Resources, Texas Tech University, Lubbock, TX, United States

**Keywords:** wide hybridization, preferential chromosome transfer, chromosome introgression, gametocidal genes, distant relatives

## Abstract

Wide hybridization is an important plant breeding strategy that can be used to expand the available genetic variation in present-day crops towards breeding for enhanced agronomic performance. The primary challenge in wide hybridization is the presence of reproductive barriers and genetic incompatibilities that limit the transfer of desirable wild or distant alleles in the genetic background of cultivated plant species. Here we provide perspectives on the possible role of hybrid sterility and gametocidal genes on the observed preferential introgression in exotic germplasm of rice. We argue that while these aberrant introgression and segregation behavior of wild or distant chromosomes presents significant barriers in exploiting ancestral germplasm in breeding, the same mechanisms can also be exploited to enhance the transfer of wild alleles in a cultivated genetic background. Understanding the genetic basis of preferential introgression and segregation in wide hybrids will have serious implications in our ability to capture ancestral genetic variation that can add significant agronomic value to staple crops like rice.

## Introduction

The end of the last ice age 10,000-12,000 years ago began humankind’s continuing pursuit to tame the wild. As vegetation flourished and humans shifted to gathering grains from large-seeded grasses, they observed that some plants of the same wild grass species have better traits than others and favored growing them every season. This cycle of artificial selection for traits that were deemed beneficial initiated the protracted process of plant domestication.

In time, domesticated plants lost many traits that were essential for their survival in the wild like seed shattering, dormancy, photoperiodicity and indeterminate habit (Meyer and Purugganan, 2013). With it, reproductive barriers and genetic incompatibilities evolved that would eventually distinguish the domesticated plant as a distinct species from its wild progenitor. Despite species boundaries however, gene flow between the domesticates and wild relatives remains possible, to varying extents (Khush and Brar, 1992).

Plant breeders have capitalized on the semi-permeable nature of species boundaries to access the genetic variation left behind in the distant relatives of crops during domestication. Using wide hybridization, or the artificial crossing between cultivated plants and their wild or distant relatives, existing gene pools of crops have been enriched and agronomic performances improved. Indeed, many success stories in plant breeding have much to attribute to novel genetic recombinations produced from wide hybridizations. In rice, the introgression in mega-varieties of the broad-spectrum bacterial blight resistance *Xa21* allele from the wild *O. longistaminata* (Khush et al., 1990) and the flooding tolerance *Sub1A* allele from the landrance FR13A (Septiningsih et al., 2009) are just two noteworthy examples of how exotic alleles can add significant value to crops. Despite these gains, there remains a tremendous amount of genetic potential in wild or distant crop relatives that is yet to be exploited for breeding purposes.

The incorporation of wild alleles in the genetic background of plant domesticates is not always straightforward. Introgressions are usually stochastic and at the same time differential, with some loci having higher propensity for introgression than others. Generally, loci with alleles conferring advantageous traits tend to introgress more frequently, whereas loci associated with reproductive isolation will introgress less (Harrison and Larson, 2014; Suarez-Gonzalez et al., 2018). Oftentimes, this manifests in the preferential retention and transmission of a specific loci from one generation to another. The nature of such phenomena has been established as a function of genomic regions as opposed to the whole genome, where chromosome segments that are consistently transmitted from one generation to another possibly carry genes that ensure its own stable transmission (Harrison and Larson, 2014). In the context of plant breeding, differential introgression creates challenges when alleles that are advantageous to the wild relative but not necessarily to the cultivated species, provide fitness advantage in the wide hybrids. Desirable trait introgression becomes more complicated when wild loci conferring deleterious traits in a cultivated background are tightly linked to traits of agronomic interests. More so when wild introgressions are regulated by genetic factors localized in the wild genome itself.

In rice (*Oryza sativa* L.), preferential retention and transmission of chromosome segments have been reported in progenies of interspecific crosses between cultivars and distant relatives. In this paper, we provide perspectives on possible mechanisms underlying the aberrant chromosome introgression in exotic rice germplasm derived from *O. nivara* and *O. latifolia* (Furuta et al., 2016; Angeles-Shim et al., 2014). We drew parallelisms between the genetic drivers of preferential chromosome transfer that have been previously reported and the observed behavior of chromosome introgressions in our rice germplasm.

## The curious patterns of preferential chromosome transfer in rice exotic germplasm

### Case 1: *O. latifolia* introgression lines

**Figure 1:**
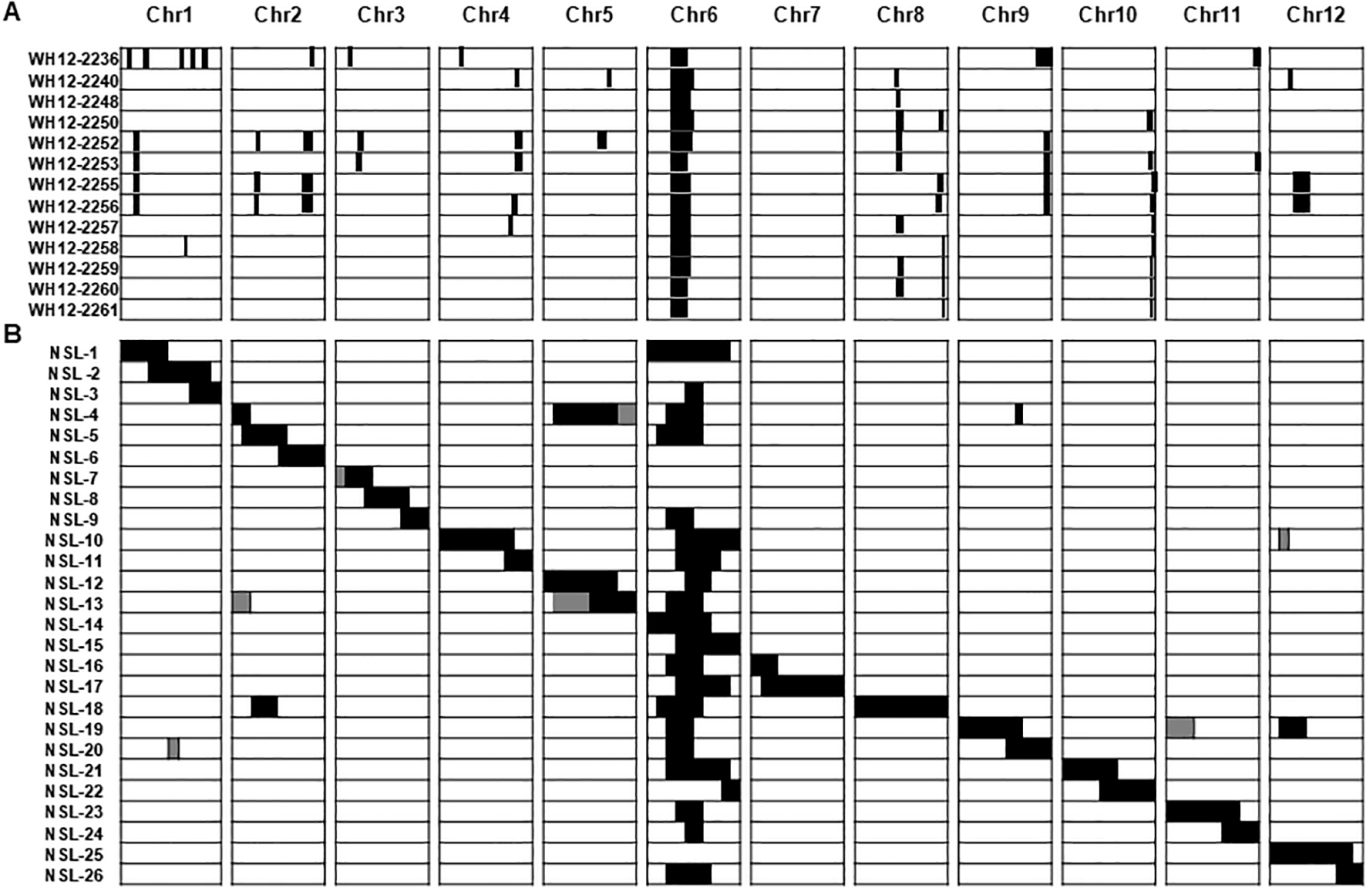
Patterns of preferential chromosome transfer in exotic rice germplasm. **(A)** Representative MDILs of *O. latifolia* in the background of *O. sativa* elite breeding line IR31917 showing stable transmission of *O. latifolia* chromosome 6 segments. Figure was adapted from Angeles-Shim et al., 2014. **(B)** Chromosome segment substitution lines of *O. nivara* in the background of *O. sativa* cv. Nipponbare (NSL) showing stable transmission of *O. nivara* chromosome 6 segments in twenty-one lines. Figure was adapted from Furuta et al., 2016. Black areas in **(A, B)** indicate wild introgression. chr, Chromosome.

Twenty-seven diploid lines that segregated from monosomic alien addition lines (MAAL) of the allopolyploid *O. latifolia* in the genetic background of the elite breeding line IR31917 were genotyped to have overlapping fragments of *O. latifolia* chromosome 6 but not a single introgression from chromosome 7 (**Figure 1A**) (Angeles-Shim et al., 2014). Mapping of the location and extent of *O. latifolia* introgression in the MAAL-derived introgression lines (MDILs) using a combination of SNPs, SSRs, STS and indel markers identified 32 wild introgressions distributed across the genome except in chromosome 7. The chromosome 6 introgression mapped between 9.42 and 22.72 Mb, with each MDIL carrying an overlapping segment that spans 9.42 Mb. Evaluation of the 27 diploid lines for a suite of agronomic traits showed no penalty on panicle fertility of the wild introgressions.

### Case 2: *O. nivara* chromosome segment substitution lines

A similar pattern of preferential transmission of chromosome 6 segment was observed in *O. nivara* CSSLs in the genetic background of the elite *japonica* cultivar Koshihikari (NSLs) (**Figure 1B**) (Furuta et al., 2016). Wild introgressions were identified using SNP markers that are evenly distributed across the genome at an average of 2.4 Mb intervals. The full *O. nivara* genome was represented in 26 NSLs, each carrying a contiguous or overlapping chromosome fragment from the wild donor. Of the 26 NSLs, 21 have substituted segments of *O. nivara* in chromosome 6. The substituted chromosome 6 segments span the full chromosome, with overlaps between 13.7 to 20.2 Mb.

## Putative hybrid sterility locus in chromosome 6 of *O. latifolia* and *O. nivara*

Hybrid sterility is a post-zygotic reproductive barrier that has been widely reported in crosses between the Asian (*O. sativa*) and African cultivated rice (*O. glaberrima*), as well as between the cultivated rice and its wild relatives (Li et al., 2020). Locus controlling hybrid sterility (*S*) functions as a gamete eliminator, aborting gametes carrying the opposite allele in heterozygotes and promoting the frequent transmission of advantageous allele at this locus (Koide et al., 2008). In rice, more than 50 *S* loci controlling hybrid sterility have been identified from various *Oryza* species and have been mapped across the different chromosomes in the rice genome (Li et al., 2020). Several studies have investigated hybrid sterility between *O*. *sativa* and *O*. *glaberrima*. Notable *S* loci such as *S19*, *S20*, *S37(t)*, *S38(t)* and *S39(t)* show a transmission advantage for the *O. glaberrima* allele (Xu et al., 2014). Even intra-specific hybrid sterility loci between subgroups of the Asian cultivated rice typically exhibit a transmission advantage of specific alleles. *S67* is a recently reported hybrid sterility locus between two subgroups of temperate *japonica* rice and basmati, demonstrating preferential transmission of temperate j*aponica* allele (Lv et al., 2024).

Chromosome 6 harbors the hybrid sterility locus *S1* and *S6* (Yang et al., 2016; Koide et al., 2008). *S1* maps in the distal end of the short arm of chromosome 6, whereas *S6* is located near the centromeric region at 18.7 Mb. The *S1* locus was first discovered in *O. glaberrima* (Sano et al., 1979), although a similar hybrid sterility locus that maps between 1.76 and 2.88 Mb in chromosome 6 have also been identified in *O. nivara*, *O. rufipogon* and *O. barthii*. Conversely, *S6* was originally discovered in crosses between *O. rufipogon* and *O. sativa* subsp*. japonica* cv. T65 (Koide et al., 2008). Like other *S* locus identified in *Oryza* species, *S1* and *S6* act as gamete eliminator, selectively favoring gametes with the wild *S* allele over those carrying the *O. sativa S* allele in heterozygotes. The control of hybrid sterility by the *S1* locus is observed in both male and female gametes, a pattern also seen with the *S6* locus (Zhou et al., 2010). The selective pressure imposed by *S6* in both male and female gametes reinforce its preferential retention and transmission in hybrid generations (Yang et al., 2016; Koide et al., 2008).

In the case of both MDILs and NSLs, the introgressed fragments from chromosome 6 of *O. latifolia* and *O. nivara*, respectively, overlap with the mapped position of the *S6* locus. This indicates the possibility of a novel hybrid sterility locus in both wild relatives that is allelic to the reported *S6* from *O. rufipogon* (*S6^rufipogon^*). Like *S6^rufipogon^*, it is possible that *S6^latifolia^* and *S6^nivara^* alleles function as gamete eliminator, promoting their preferential transmission and retention in the *O. latifolia* MDILs and *O. nivara* NSLs. Whether the hybrid sterility locus occurs in either one or both gametes is yet to be determined.

Incidentally, similar patterns of preferential transfer of chromosome 6 segment carrying the *S6* locus have not been observed in other CSSLs developed using *O. barthii*, *O. longistaminata*, *O. rufipogon and O. glaberrima* (Bessho-Uehara et al., 2017; Ramos et al., 2016; Furuta et al., 2014; Angeles-Shim et al., 2010). To date, the *S6* locus has only been identified in *O. rufipogon* and putatively in the *O. nivara* NSLs and *O. latifolia* MDILs. This contrasts with the reported microco-linearity of other hybrid sterility locus like the *S1* in at least 4 *Oryza* species with AA genome (Yang et al., 2016). To determine the conservation of the *S6* locus in *Oryza* species with AA genome, syntenic analysis was carried out for *O. sativa* cv. Nipponbare, *O. rufipogon*, *O. barthii*, *O. glaberrima* and *O. nivara* chromosome 6 using data from the rice genome hub (https://rice-genome-hub.southgreen.fr/synvisio) (Bandi and Gutwin, 2020).

**Figure 2:**
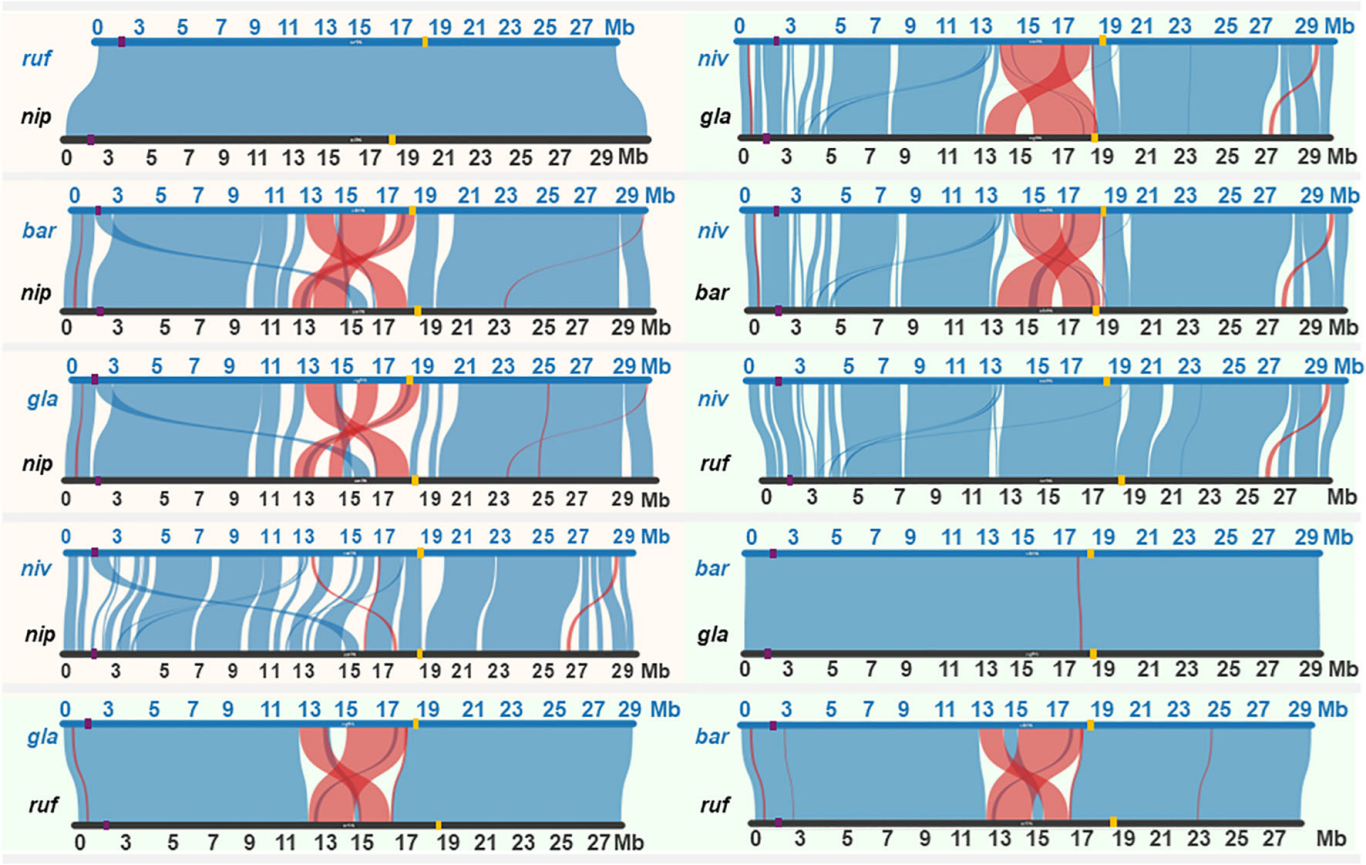
Synteny across chromosome 6 of wild rice species with AA genome. Orange shaded region highlights synteny between the cultivated *Nipponbare* (*nip*) and its wild relatives, while the green shaded region illustrates synteny among the wild species: *O*. *barthii* (*bar*), *O*. *rufipogon* (*ruf*), *O*. *glaberrima* (*gla*), and *O*. *nivara* (*niv*). Purple and yellow bars on chromosome represent the position of *S1* and *S6* locus, respectively.

Syntenic analysis revealed variations in the structural arrangement of chromosome 6 across the four *Oryza* species (**Figure 2**). *O. rufipogon* maintained complete synteny with *O. sativa* cv. Nipponbare, while both *O. barthii* and *O. glaberrima* exhibited large-scale inversion events in the centromeric region for chromosome 6. *O. rufipogon* also shared synteny with *O. barthii* and *O. glaberrima* across chromosome 6 except for the centromeric region. The lack of synteny between *O. rufipogon*, *O. barthii* and *O. glaberrima* suggests that the *S6* locus may be absent or transcriptionally inactive in the latter two, which would explain the normal chromosome transmission in previously reported *O. barthii* and *O. glaberrima* CSSLs (Bessho-Uehara et al., 2017; Angeles-Shim et al., 2010). Incidentally, the lack of systematic transmission of chromosome 6 segment in the *O. rufipogon* CSSLs developed by Furuta et al. (2014) contradicts with the original discovery of the *S6* locus in another accession of *O. rufipogon* (Koide et al., 2008). This may be due to genetic background effects similar to what has been reported for the *S24* sterility locus where male semi-sterility was only observed in *S24* heterozygote in a *japonica* background but not in an *indica* background (Kubo et al., 2011).

*O. nivara* displayed minor syntenic disruptions at the centromeric region compared to cv. Nipponbare and showed inversion events at the centromeric region between 13 and 17 Mb relative to *O. barthii* and *O. glaberrima* but not *O. rufipogon*. Notably, *O. nivara* and *O. rufipogon* showed synteny at the *S6* locus, supporting the proposed presence of a functional *S6* locus in *O. nivara* that could have facilitated the stable inheritance of the wild chromosome 6 segment in the NSLs. Unlike *O. rufipogon* however, *O. nivara* exhibits unique structural variations compared to *O. sativa* that can potentially harbor genes or regulatory elements that enhance its PCT potential when combined with the functional *S6* locus. We hypothesize that the interplay between the *S6* locus and these distinct genomic features in chromosome 6 of *O. nivara* drives its heightened propensity for PCT. In the presence of linkage, the *S6* locus will be inherited together with other proximal loci. With generation advance, distortion in transmission ratio exerted by *S6* would extend beyond the locus itself, favoring the transmission of overlapping chromosome 6 segments in advanced progenies. In the development of the NSLs, it appears that the selective advantage imparted by the *S6* locus in fact led to the accumulation of overlapping segments on chromosome 6 despite repeated backcrossing. The consistent retention of these overlapping segments, driven by the transmission bias of the *S6* locus could effectively integrate *O. nivara* alleles across chromosome 6 in the Koshihikari background.

*In silico* analysis of the annotated genes within the 13-18 Mb of the introgressed chromosome 6 segment of *O. nivara* identified genes involved in fatty acid metabolism and cell death regulation, both of which have been reported to play crucial roles in pollen viability and gametophyte competitiveness under varying environmental conditions (Zhang et al., 2021; Wang et al., 2024). For example, fatty acid metabolism is necessary to maintain cell membrane integrity, which can support pollen resilience and function. The regulation of cell death within this region might contribute to selective elimination processes under competitive fertilization, promoting the preferential transfer of certain chromosome segments (Koide et al., 2008).

## Genetic regulation of chromosome distortion and spikelet fertility

The MDILs showed normal spikelet fertility whereas two out of the 26 NSLs (nos. 16 and 17) expressed approximately 50% reduction in the number of grains per panicle. Both NSLs have overlapping, substituted segments in chromosome 6 and 7 of *O. nivara*. However, phenotype-genotype association using the full NSL set putatively associates the spikelet fertility reduction in lines 16 and 17 with the 3.9–7.9 Mb introgressed segment from chromosome 7 of *O. nivara*.

The observed fertility reduction in NSLs 16 and 17, marked by a 50% decrease in grain number per panicle, is likely linked to a gametocidal-like gene (Gc)-like. Gametocidal gene systems, as seen in hybrids of *Triticum aestivum* and *Aegilops sharonensis*, typically involve two elements: a “breaker,” which induces DNA breaks in gametes lacking specific chromosomal segments, and an “inhibitor,” which mitigates the breaker’s effects to preserve gamete viability (Knight et al., 2015). However, in the case of NSLs 16 and 17, there appears to be no evidence of an inhibitor-breaker like element, suggesting that the observed fertility reduction may arise from a gene(s) locus/loci acting independently.

Gene ontology (GO) analysis of the chromosome 7 region in NSL 16 and 17 revealed several pathways potentially contributing to reduced fertility. Key genes associated with lipid metabolism, cell wall biosynthesis, and DNA damage responses were identified, indicating the presence of mechanism that are associated with gamete stability and viability. For instance, the gene *ONIVA07G13230.1*, which encodes a *fatty acyl-CoA reductase* essential for lipid biosynthesis, likely plays a critical role in maintaining membrane stability and pollen viability (Zhang et al., 2021; Wang et al., 2024; Yang et al., 2017). Disruption of this pathway could lead to unstable pollen and selective gamete loss. Similarly, genes involved in cell wall biosynthesis, such as those encoding mannan synthase and cellulose synthase, are crucial for maintaining the structural integrity of pollen cell walls. Weakened cell walls in gametes lacking the protective chromosome segment may render them more susceptible to environmental and metabolic stress, further reducing fertility.

In addition to structural and metabolic disruptions, genes linked to DNA degradation and hypersensitive responses appear to exacerbate gamete instability. GO terms associated with exonuclease activity and defense responses suggest that DNA damage pathways may be hyperactivated in gametes without the chromosome 7 segment, leading to selective cell death. Furthermore, genes encoding AAI domain-containing proteins and *AP2/ERF* transcription factors within the introgressed region may influence pollen development and stress responses, indirectly affecting fertility (Long et al., 2023; Zhang et al., 2024). These proteins regulate lipid transport and developmental processes critical for gamete viability, and their altered function in NSLs 16 and 17 could explain the observed fertility reduction.

The observed 50% reduction in spikelet fertility is not mutually exclusive to the breaker-inducer complex as mentioned above. Our findings align with discoveries in other studies where reduced pollen viability significantly impacts spikelet fertility. This is well-illustrated in the reproductive behaviors associated with the *S6* locus in rice hybrids (Koide et al., 2008). Histological analyses of female gametogenesis in *S6^rufipigom^/S6^sativa^* heterozygotes demonstrated that nearly half of the embryo sacs exhibited structural abnormalities. This aligns with the 50% fertility reduction observed in affected hybrids. While male gamete dysfunction is not visually apparent at the pollen grain stage, genetic evidence strongly suggests post-pollination competition or incompatibility, further exacerbating fertility losses. The absence of viable *S6^sativa^* gametes in progeny confirms the decisive role of selective gamete abortion mediated by the *S6^rufipigom^* locus. However, in our findings we can see a reduction of spikelet fertility under a homozygous state. Nonetheless, we can assume that the introgressed region of chromosome 7 (NSL 16 and NSL 17) might harbor a functional gene that directly leads to the 50% reduction in spikelet fertility in these NSLs.

The absence of an inhibitor element in this system suggests a simpler mechanism than the classic Gc model, where the breaker-like locus alone drives selective gamete loss. This fertility distortion aligns with the preferential transmission of the chromosome 7 segment and may provide a selective advantage in specific contexts. The cumulative evidence points to a multifaceted mechanism of fertility reduction involving disruptions in cell wall integrity, lipid metabolism, and DNA stability. Further research is necessary to identify the specific genetic contributors within the substituted chromosome 7 segment and to elucidate how these genes interact to influence fertility in hybrid genetic backgrounds. Understanding these mechanisms could have significant implications for introgression breeding and the regulation of hybrid fertility.

Exotic germplasm offers significant potential for plant improvement, but its use in breeding is often constrained by the biased transmission of distant genomic regions. From an evolutionary perspective, this biased transmission where one genotype is favored over another at the expense of overall fitness may seem paradoxical. This concept mirrors the early misconceptions about non-coding DNA, which was once considered “junk” but is now recognized for its crucial role in regulating gene expression. Similarly, this PCT associated with sterility loci can be exploited as a mechanism to access novel genetic variations from wild or distant crop relatives, rather than being viewed as a barrier for beneficial wild introgressions. By manipulating hybrid sterility genes, we can efficiently introduce these novel alleles into breeding programs without compromising their stability. The sterility locus ensures the retention of viable gametes, although its heterozygous state often leads to reduced gamete viability.

However, if the sterility locus is tightly linked to genes that negatively impact agronomic traits, we may need to develop strategies to mitigate these effects. While no such complications have been observed in studies involving sterility loci to date, it remains essential to understand the genetic mechanisms controlling these loci. Such knowledge will be crucial for integrating distant relatives into breeding programs, ultimately helping to develop more robust rice cultivars with broader genetic diversity.

## Data Availability

The raw data supporting the conclusions of this article will be made available by the authors, without undue reservation.
